# Popular health surveillance in the face of climate emergencies

**DOI:** 10.1371/journal.pgph.0006978

**Published:** 2026-08-05

**Authors:** Fernando Ferreira Carneiro, Graciela Stornini de Almeida, Teresinha Pereira da Silva, Soraya Vanini Tupinambá, Vera Lúcia de Azevedo Dantas, Vanira Matos Pessoa, Maria Rocineide Ferreira da Silva, Tania Maria Peixoto Fonseca

**Affiliations:** 1 Fiocruz Ceará, Eusébio, Ceará, Brazil; 2 Landless rural workers’ movement - MST - Settlement Santa Rita de Cássia II, Nova Santa Rita, Rio Grande do Sul, Brazil; 3 Indigenous Leadership Movimento Potigatapuia - Aldeia Mundo Novo, Monsenhor Tabosa, Ceará, Brazil; 4 Health Minister and Universidade Estadual do Ceará, Fortaleza, Ceará, Brazil; 5 General Coordination of Health Surveillance and Reference Laboratories, Fiocruz, Rio de Janeiro, Brazil; Fundacao Oswaldo Cruz, BRAZIL

## Abstract

Public health emergencies associated with extreme weather events – such as floods and droughts – have intensified, revealing the complex relationship between climate emergencies and structural inequalities. In Brazil, rural populations, riverine and forest communities, Indigenous peoples and those living in urban peripheries are disproportionately exposed to the impacts of climate emergencies, which exacerbate socio-environmental vulnerabilities. To protect populations, community-driven initiatives known as “Popular Health Surveillance” (PHS) have emerged and gained political relevance. PHS integrates popular knowledge, scientific understanding, and perspectives from health systems. Anchored in the principles of Popular Education in Health, the ecology of knowledge, and the Social Determination of Health and Good Living (Bien Vivir), PHS recognizes, values, and combines scientific knowledge, popular knowledge, and territorial experiences, promoting intercultural dialogue as a core methodological approach. In the context of climate emergencies, a major challenge for health systems is the implementation of effective mitigation, prevention, and resilience strategies – efforts that require coordination and collaboration among multiple actors and meaningful participation of populations in higher-risk areas. This article presents a conceptual framework and approaches for implementing Popular Health Surveillance in the context of climate emergencies. A documentary study identified national and international experiences of participatory health surveillance and examined them in dialogue with the PHS proposal. These experiences were analyzed based on their definitions and main operational strategies. PHS was further examined through its theoretical principles, strategies, attributes, and application in the face of climate emergencies. PHS is not only a participatory monitoring tool, but a strategy for promoting socio-environmental, cognitive, and health justice. In the context of intensifying climate crises, this approach is essential strengthening community adaptive capacity, integrating diverse knowledge systems, and demanding more inclusive and democratic public policies from an emancipatory perspective.

## 1 Introduction

The climate crisis is one of the greatest threats to public health in the 21st century. By contributing to the wider spread of respiratory, infectious, and chronic conditions, prolonged droughts, catastrophic floods, heat waves, and other extreme weather events overwhelm health systems and exposes gaps in institutional preparedness and response efforts [1,2]. Rural populations, riverine and forest communities, Indigenous peoples, and those living in urban peripheries of the Global South are disproportionately vulnerable to climate risks due to social, economic, environmental, commercial, and political determinants that shape both their exposure to and resilience against extreme weather events. The impacts of climate emergencies deepen these socio-environmental vulnerabilities – including land and water access, territorial security, environmental contamination, institutional racism, and fragile health and social protection systems – and reveal the intricate relationship between climate change and structural inequities. For example, the 2023 report of the Intergovernmental Panel on Climate Change (IPCC) highlights that smallholder farmers and fishing communities – particularly Indigenous and traditional peoples – are among the groups most vulnerable to climate related risks [3].The report demonstrates that these risks are compounded by persistent historical inequalities, intensified by intersectional factors such as gender, ethnicity, and income. Direct consequences of climate emergencies include food insecurity, the proliferation of vector-borne diseases, forced migration, increasing impoverishment, and rising levels of violence [3,4].

For many Indigenous and traditional peoples whose ways of life, local economies, and everyday practices are grounded in an understanding of human existence as interdependent with waters, forests, and fauna [5–7], disruptions to the local environment also mean disruptions to their knowledge systems and wellbeing. At the turn of the twenty-first century, as environmental conflicts intensified, these disruptions catalyzed numerous social movements among populations affected by agribusiness, hydrobusiness, mining, and industrial expansion. In response, communities have developed and led territorial initiatives to analyze and mitigate the health impacts of territorial transformations, ecosystem degradation, pollution, and the increasing precarity of their livelihoods. These collective actions highlight how community-driven mobilization, organization, and resistance are essential in the face of growing climate-related risks to health [8–12]. There is a need for evidence to inform and strengthen these processes; therefore, in this paper – part of a collection of articles on the governance of national public health agencies – we examine an example of community-driven knowledge generation and surveillance in Brazil.

In Brazil, initiatives referred to as “Popular Health Surveillance” (PHS) have gained prominence by empowering communities as protagonists in the defense of life, integrating local knowledge with scientific understanding and policy perspectives to monitor, prevent, and mitigate the impacts of climate change on collective and ecosystem health. These initiatives, grounded in dialogical and participatory processes, generate critical knowledge for decision-making, action and the reorientation of institutional health practices [13–18].

In this context, the term “Popular” refers to approaches rooted in critical thinking and social movements, such as the concept of “Popular Education” by Paulo Freire, the Brazilian pedagogue who formulated the “Pedagogy of the Oppressed” [19]. In contrast to top-down health monitoring and response approaches that perpetuate historical injustices [20], PHS emerges as a strategic and transformative framework based on community protagonism and active participation. By recognizing and strengthening popular and traditional knowledge and integrating it with scientific and technical expertise and local practices, PHS enables: i) the construction of territorialized, culturally appropriate, integrated and sustainable solutions; ii) strengthened community resilience in the face of multiple contemporary crises; iii) the promotion of environmental justice; and, iv) the integration of public policies and local actions [15]. These initiatives, developed within territories through community organization and emancipatory action research, often face invisibility imposed by the institutional structures of State Health Surveillance [16].

Therefore, the development, expansion, and institutionalization of participatory instruments, strategies, and actions for disaster mitigation, prevention, and resilience—integrating multiple knowledge systems and emphasizing the engagement of local populations in risk areas—constitute one of the central challenges for the governance of health systems, particularly in response to climate‑related public health emergencies.

### 1.1 Context of the Brazilian health system

Brazil is a continental country in South America, with a population of 213.4 million inhabitants, organized as a federation with 27 states and 5,569 municipalities [21]. Since the Federal Constitution of 1988, health has been recognized as a universal right and a duty of the State. The Unified Health System (SUS) is founded on the principles of universality, comprehensive care, equity and social participation. It is a decentralized, operationalized, and regionalized system shared among the federal, state, and municipal governments, with services organized by levels of complexity (primary, secondary, and tertiary care). At the federal level, the Ministry of Health (MS), at the state level, the State Health Secretariats, and the municipal Health Secretariats, form collegiate management bodies of SUS. In dialogue with the municipal, state and national Councils, these bodies define the public health priorities across federation. In addition, social participation and the definition of national SUS guidelines occur through municipal, state and national health conferences, where priorities are deliberated every four years with broad societal participation. This governance structure enables allows for more locally tailored health policies and promotes shared responsibility and community engagement. Public health strategies are agreed upon at all three levels of government through formal mechanisms, such as the Tripartite Interagency Commission (CIT). These collaborative platforms support more coherent planning, equitable financing, and alignment with national priorities. Despite challenges such as funding gaps, infrastructure limitations, and regional disparities, the SUS remains a global reference in public health policy and a symbol of Brazil’s commitment to social justice in health [22].

Within this context, the Oswaldo Cruz Foundation (Fiocruz) plays a strategic role. Linked to the Ministry of Health, Fiocruz is a leading institution in science, technology, and innovation in health. It operates with budgetary autonomy and democratic governance, with its president and directors elected and guided by deliberative councils. Fiocruz comprises 16 technical-scientific units and six regional offices, working in areas such as education, research, innovation, health care, surveillance, communication, and technological development. Internationally recognized, it integrates scientific research with the production of essential health inputs, contributing to the strengthening of the SUS and the promotion of equity and human dignity [23].

The Ministry of Health maintains a Committee for Monitoring Events in Public Health, in which Fiocruz participates. These meetings monitor potential outbreaks of national interest and track issues of international relevance. Emergency Operations Centers (COE) are activated during public health emergencies and Fiocruz plays an active role by providing technical and scientific expertise, producing medical inputs (such as vaccines and medicines), training human resources, supporting diagnostic and contributing to assistance, teaching, research and innovation [24].

To better respond to future emergencies, countries are strengthening institutions such as Fiocruz. In response to the need for evidence to support these improvement processes, the Alliance for Health Policy and Systems Research and the WHO Health Emergencies Programme supported research in eleven countries to identify strategies to strengthen the governance of Public Health Institutes in this area. During 2024 and 2025, Fiocruz contributed to this initiative by generating evidence to assess whether Popular Health Surveillance can strengthen health services in the face of climate emergencies. This paper is part of that research effort and seeks to present a conceptual framework and approaches for implementing Popular Health Surveillance in the context of climate emergencies.

## 2 Metodology

This is a scoping review [25] guided by the research question: “How can Popular Health Surveillance serve as a strategy to strengthen the actions of health systems in response to public health emergencies associated with climate change?”

The literature search was conducted in the SciELO, Web of Science, Google Scholar, and Virtual Health Library databases. The following descriptors were used, combined through the Boolean operators “AND” and “OR”: “Popular Health Surveillance” OR “Civil Surveillance” OR “Indigenous Surveillance” OR “Workers’ Health Surveillance” OR “Community-Based Surveillance” OR “Citizen Science-Based Surveillance” OR “Critical Epidemiology” OR “Popular Epidemiology” OR “Sociocultural Epidemiology” OR “Community Epidemiology.” Combinations were performed using the AND operator to associate terms and refine the search, and the OR operator to broaden the scope of results.

The following inclusion criteria were applied: publications explicitly addressing participatory or community-based health surveillance practices; studies discussing the relationship between social participation and health surveillance; publications between 1960 and 2025; scientific articles, books, book chapters, technical reports, and institutional documents considered seminal or reference works; and texts published in Portuguese, English, and Spanish. A total of 30 emblematic publications were selected. For the purposes of this study, “emblematic” refers to seminal works and contributions by key authors in the field that explicitly describe their surveillance modality and its relationship with social participation in health. Following the selection of these 30 publications, spanning from the 1960s to 2025, a full-text reading was conducted.

The participatory surveillance modalities were analyzed in terms of their conceptual foundations, operational strategies, and emancipatory character. Based on this analysis, a concept of Popular Health Surveillance was developed, including its attributes, principles, strategies, and application in the context of climate-related emergencies. The concept of Popular Health Surveillance, which constitutes the central focus of this study, is currently under theoretical development.

## 3 Results and discussion

### 3.1 Conceptual basis for Popular Health Surveillance

The hegemonic model of surveillance, rooted in nineteenth and twentieth-century conceptions of health, maintains an approach centered on disease control and shaped by a warmongering perspective, is expressed in metaphors such as the “war against microbes.” This structure – reinforced by militarized terminology such as “surveillance,” “control,” “sentinel event,” “campaign” - operates predominantly through in the monitoring of sick or suspected individuals sometimes resulting in an authoritarian sanitary practice that marginalizes popular participation [26]. Even during the Covid-19 pandemic, the Brazilian State preserved this surveillance paradigm, revealing persistent difficulties in providing institutional responses that incorporate more democratic and participatory approaches.

At the international level, particularly around the turn of the century, Latin American experiences - such as Critical Epidemiology, Community Epidemiology, Sociocultural Epidemiology, Workers‘Health Surveillance and Civil Surveillance - emerged as participatory and counter-hegemonic approaches to Surveillance, marked by its democratic, participatory, and critical orientation. At the same time, in the United States, Popular Epidemiology was developed, also drawing inspiration from this broader history of community‑based approaches. These initiatives have strengthened community organization in the face of diverse threats to populations health, reinforcing movements for health and environmental justice. In another trajectory supported by the WHO in African countries, community-based surveillance has played a strategic role in expanding participation networks within surveillance systems. Following the Covid-19 pandemic in Brazil, new modalities of surveillance have emerged, such as Popular Health Surveillance and Indigenous Surveillance [16]. The Table 1 presents a summary of these approaches.

**Table 1:** Types of participatory surveillance (emblematic examples).

Participatory Surveillance Modality	Summary definition	Key operational strategies
Popular Health Surveillance [8,11–13,15–18,27–29]	Popular protagonism in the defense of life from an emancipatory perspective, involves the autonomous production of data with community control over the entire process, the use of social technologies, popular communication and a meaningful dialogue of knowledge with academia and health systems.	Action Research. Participatory Mapping. Culture circles. Conversation circles. Social technologies. Photovoice. Participatory monitoring. Workshops. Exploratory/experiential visits in the territories.
Civil Surveillance [14,26,30,31]	Civil health monitoring developed by popular sectors in collaboration with professionals committed to the proposal. It emphasizes popular participation and fosters dialogue between scientific knowledge and community knowledge.	Popular Education. Participatory diagnosis. Community epidemiology. Alternative research practices carried out by health and education professionals in collaboration with organized popular sectors of civil society.
Indigenous Surveillance [32,33]	It is a surveillance and monitoring strategy for the protection of indigenous territories, which is essential for ensuring the conservation of forest, biodiversity, mitigating the impacts of climate change and safeguarding peoples themselves.	Independent territorial monitoring (including geospatial methods). Technologies appropriated and managed by Indigenous communities. Participatory cartographies. Dialogue of knowledge.
Workers’ Health Surveillance [34,35]	It is a set of continuous and systematic actions, part of health systems, which aims to promote health, prevent diseases and accidents, and reduce risks in the workplace. The participation of workers in the process is one of its principles.	Participatory risk mapping. Tree of causes. Notifications of participative accidents and injuries. Intersectoral commissions with social participation. Participative inspection of work environments.
Community-Based Surveillance [36–38]	Systematic detection and reporting of events of public health importance within a community-by-community members. It is a health surveillance tool that draws on popular knowledge and the engagement of community leaders to identify risks early and, in partnership with local health units, trigger rapid responses.	Simplified case definitions and reporting forms submitted to health facilities for verification, investigation, data collection, analysis and response. Community Leadership Training. Participatory cartographies. Adapted communication materials.
Citizen Science-Based Surveillance [39,40]	Participatory Surveillance model in which ordinary citizens collaborate with scientists and government agencies in the collection, monitoring, and analysis of data to generate scientific knowledge and support public policies.	Voluntary participatory monitoring. Records of ecological patterns of species, spread of infectious diseases, population trends, landscape changes and climate changes. Use of accessible technologies such as Mobile Applications and Digital Platforms: which facilitate the registration of photos, geographic location (GPS) and real-time data. Feedback for those involved.
Critical Epidemiology [41–43]	It proposes that health and disease are not merely isolated statistical or biological events, but processes shaped by social, economic, and political structure. This perspective the traditional and purely biological view of diseases and instead centers the social determination of health.	Participatory Monitoring. Strategic Planning. Social Control.
Sociocultural Epidemiology [44]	An interdisciplinary dialogic proposal, it seeks to contribute to the analysis of health problems through the participation of diverse social actors and the use of theoretical and practical tools, developed in the social sciences, biomedical fields, humanities and the arts. It integrates biological, social, symbolic, emotional and cultural dimensions, while recognizing scientific and non-hegemonic forms of knowledge.	Risk scenarios. Avoidable damage. It integrates and articulates quantitative and qualitative methods, using epidemiological indicators in dialogue with narratives, ethnographies, life histories and other strategies that make it possible to understand the social meaning of becoming ill.
Community Epidemiology [45]	It is a way of thinking and practicing medicine that takes community as its central subject, aiming to transform the living conditions life and everyday contexts activities where the in which disease is produced. Through a critical and collaborative process, the Community and “health operators” work together to progressively overcome health problems.	Community training. Building sensitive indicators. Decodable language. Developing participatory diagnoses and programs.
Popular Epidemiology [46,47]	An approach which local communities and lay people actively participate in the production of scientific knowledge on health risks, drawing on perspectives from Citizen Science, Environmental Justice and Critical Epidemiology. It mobilizes community members around the goal of identifying and mitigating environmental stressors and local disease patterns.	Participatory research protocols. Community-based research. Social mobilization. Partnering with scientists and activists. Diaries and health “lay” investigations.

Surveillance initiatives that promote social participation are fundamental to expanding the reach of health actions. However, recent Brazilian experience demonstrates significant differences between Popular Health Surveillance and other participatory modalities, such as Community-Based Surveillance or Citizen Science-based Surveillance. What distinguishes PHS is its essentially emancipatory character, an aspect that has been systematically analyzed in academic research in the country [8,9,13–15,17,18,24–26,30,40]

Popular Health Surveillance can be defined as popular protagonism in the defense of life, where local populations take collective ownership of the entire surveillance process, from the production and analysis of data to the implementation of concrete actions using social technologies of participatory monitoring (e.g., participatory mapping community health logs, territorial walks, community assessment tools, risk calendars, etc.); popular communication (e.g., local radio, community art, community alerts, story-telling, community assemblies, etc.); and generation of shared knowledge, integrating local knowledge, scientific understanding, and rights-based policy perspectives [12,15]. PHS achieved prominence by consolidating itself as an effective response to state limitations in the implementation of adequate public policies. Participatory tools stand out as innovative strategies, such as: strategic use of social networks to denounce rights violations; popular sanitary barriers; popular and academic observatories; and local committees organized by indigenous peoples, quilombolas, fishermen, and family farmers [16]. It is important to highlight that Popular Health Surveillance does not seek to replace the State, but requires instead that the State fulfills its role in guaranteeing the right to health through effective public policies. In many cases, due to competing corporate and political interests, the State fails to fulfill its role in surveillance and care actions. Popular Health Surveillance acts as a necessary counterpoint, pressuring the government to assume responsibilities and strengthening health systems in their different spheres of control and action, [28].

This modality of surveillance materializes the historical demand for greater social participation in health surveillance processes, as provided for in Brazil’s National Health Surveillance Policy (2018) [48]. Its emergence as a mechanism for alerts and community mobilization intensified in 2020, in a context of growing vulnerability of traditional and peripheral populations, configuring itself as an instrument to guarantee the right to health, an environmental protection mechanism, and a strategy for the defense of life in threatened territories. More recently, PHS was cited in nine guidelines from the 17th National Health Conference, held in 2023 in Brasilia, a crucial event for the definition of public health policies in Brazil [30].

This approach represents an innovative paradigm in collective health, which can contribute to the democratization of the health system and to enhance the territorialization of public policies. It can also contribute to health, environmental, social and cognitive justice by promoting emancipatory actions. It is important to highlight that there are differences between the concepts of public health and collective health. Public health seeks to technically organize the control of health problems, while collective health seeks to politicize, historicize, and socially transform the processes that produce health and disease [49]. It is also important to highlight that it is not possible to achieve global social justice without justice in the field of knowledge, since social, political, and economic inequalities are inseparable from inequalities in the production, validation, and circulation of knowledge [50].

By integrating diverse knowledge and strengthening community participation, the PHS is configured as a powerful instrument to address inequalities and build more adequate responses to the health needs of populations, with the denunciation of violations and the formulation of public policies based on the realities experienced in the territories. In this sense, it acts as a mediation between guaranteed rights and rights effectively accessed, strengthening popular participation and social control [30,43,49,51–55]. Table 2 presents a summary of the proposed attributes for Popular Health Surveillance.

**Table 2:** Attributes of Popular Health Surveillance [15].

Popular protagonism in the struggle in defense of life.	Popular organizations and collectives act in an emancipatory perspective in the face of life-threatening situations.
**Data production.**	Popular control of the surveillance process, from the production and analysis of data to the implementation of concrete actions.
**Production of shared knowledge.**	Generation of knowledge that dialogues with popular, academic, and health service knowledge.
**Participatory monitoring using social technologies.**	Creation and use of accessible and socially appropriate technologies for the generation of territorialized data in a participatory and democratic way.
**Desirable articulation with health systems and other public policies, academia, popular organizations and networks of social movements.**	Synergies through the articulation between health services, universities/research centers and networks of social/popular movements, to enhance the action of each actor and social participation in the processes.
**Popular Communication.**	Production of information committed to the emancipation and transformation of reality through a mobilizing, dialogical and horizontal dialogue.
**Demand for rights and public policies.**	Tension for the State to fulfill its role of guaranteeing the right to health through effective public policies.

One of the attributes of community-based social work (PHS) that can enhance its actions is the “desirable articulation with health systems and other public policies, academia, popular organizations, and networks of social movements.” When the community generates information in partnership with universities, from the perspective of the ecology of knowledge, greater density of technical and political impact is gained because the data become more robust and offer evidence for action by both society and the State, often in conflict situations. PHS experiences show that academic studies conducted in isolated territories, without contributing to popular organization and without articulation with the health system, have more limited results [9,12,13,15–18,26,28–30,36].

In this sense, there are also cases in which the health system is constrained to carry out its actions in the face of local political contexts [8]. The role of the PHS is fundamental in claiming public policies in the face of the impacts of a climate event or polluting enterprises, as it reinforces the need for health systems to fulfill their role in guaranteeing the right to health of populations. In the Brazilian case, there has been a recent advance on the part of the Unified Health System, which included Popular Surveillance as part of the Health Surveillance axis of its great climate adaptation program – ADAPTASUS (USD 2 billions 2025–2035) [56]. This inclusion in public policies can contribute to the enhancement of PHS experiences in the face of climate emergencies as this approach is recognized by the health system.

### 3.2 Strategies for the implementation of Popular Health Surveillance in the context of strengthening community actions and the governance of health services

The choice of methods to build PHS strategies presupposes participatory approaches. This means that those who live in the territories can bring their knowledge in the identification and problematization of challenging situations as well as in the formulation of actions aimed at overcoming them. In doing so, they become the protagonists of these processes. Popular or community and grassroots organizations also bring valuable ideas and possibilities through their experiences. Table 3 summarizes this theoretical and methodological accumulation, in the context of a modality of surveillance that is still emerging.

**Table 3:** Synthesis of theoretical principles and strategies for carrying out Popular Health Surveillance.

Surveillance	Concept	Theoretical-practical principles	Strategies	Application related to climate emergencies	Reference texts
Popular Health Surveillance	It is the popular protagonism in the defense of life where populations take control of the entire surveillance process, from the production and analysis of data to the implementation of concrete actions using social technologies of participatory monitoring; popular communication; generation of shared knowledge, mediated by the articulation between local knowledge, academics and health services and in the claim for rights and public policies [17].	Emancipation. Popular Education. Ecology of Knowledge. Bien Vivir (Living well) Social Determination of Health/Critical Epidemiology. Intersectionality. Social participation. Solidarity. Territory	Action Research. Participatory Mapping. Popular communication. Photovoice. Participatory action plans. Participatory monitoring. Workshops. Conversation circles. Territorial experiences. Crop circles. Popular Indicators. Popular Researchers. Expressions and languages of art and popular culture. Community and solidarity economy network. Observatories and academic/popular panels.	Mining. Steel. Agribusiness. Pesticides. Air pollution. Covid 19 syndemic. Wind farms. Shrimp farming. Water and air contamination. Socio-environmentally vulnerable urban areas, indigenous and traditional territories.	Popular Health Surveillance – concepts and references [11,15,27,36,37]. Civil Surveillance [14,26,31]. Popular Health Surveillance reviews [17,36].

In addition to the Popular Health Surveillance strategies summarized in chart 3, we also highlight the development of indicators, used to guide decision-making in the face of emergencies. Grounded in the social determination of health and by Critical Epidemiology [43] the indicators are organized across a hierarchy of complexity, ranging from the individual level to lifestyle, environment or territory, and broader society. From a dialectical perspective, [43] each layer of health determination includes both indicators that express “threats to life” and those that reflect “promotion of life.” These indicators largely have a qualitative character and can be monitored by the community in the territory itself [57].

Popular health surveillance can contribute to addressing extreme weather situations through collective action. One of the results of this process is to move people from the situation of complaints to the announcement of possibilities aimed at building strategies that refer to overcoming these situations. Paulo Freire named this action as the construction of unprecedented viable (Inédito Viável) [30].

The PHS has operated as an early risk identification tool for Climate Emergencies, using territorially-based indicators (such as water scarcity, water contamination, biodiversity loss, psychosocial impacts, and food insecurity), and as an instrument for collective organization to build adaptive responses, including contingency plans, community health brigades, accessible warning systems, and social technologies for living with drought and floods [8,12,15,17,18].

Another methodological innovation in Popular Health Surveillance studies is the incorporation of Popular Researchers in the Participatory Action Research process. Their relevance is evaluated in terms of whether they: 1) contribute to territorial engagement and know their community, its histories, and geographies, 2) strengthen community bonds and cohesion by promoting practices such as participation, autonomy, popular democracy, agroecology, and care for life, 3) help to problematize the dialectical process of knowledge construction by bringing different perspectives of reality and worldviews, facilitating the exchange of knowledge, and 4) facilitate the mediation of scientific knowledge, tools, and technologies, so that they are at the service of the transformation of the community itself [12]. The work of popular researchers has been facilitated by greater ease of access to technologies that have become cheaper, such as the use of applications on cell phones, drones, portable meters or low-cost weather and environmental conditions [32,38,40].

These constructions are historical and collective, shaped by the struggle for rights, bonds of trust, and solidarity, which are renewed over time. The incorporation of Popular Researchers, in this sense - in processes of construction and transformation - promotes the democratization of spaces, mobilization, organization, as well as the popular participation of groups, communities, and individuals in processes that, together with academia, seek alternatives for the transformation of reality through the mediation of scientific knowledge. The participation of these actors in the participatory action process, as well as the exchange of experiences, struggles, reflections, and challenges, promotes the strengthening of territories, transforming and democratizing in practice, the networks of solidarity, sovereignty, sustainability, and security. This reinforces existing networks, the social fabric, and social transformation.

The methodologies developed in various experiences such as Emancipatory Participatory Action Research, Territorial Experiences, workshops, conversation circles, photovoice, culture circles, mapping and participatory action plans, strategic and independent community monitoring, observatories and popular academic panels, and community networks of solidarity economy – are examples of flexible tools, adaptable to different contexts. These techniques can contribute as valuable tools to identifying risks to the health of communities while simultaneously producing indicators that facilitate the development of preventive and adaptive actions by the government [8,11,12,15–18,27,31,33,35,42,44,46,47].

The knowledge held by communities - such as the knowledge of natural cycles, the herbs that promote health, the production of healthy food, the technologies for living with drought, the solutions found in the face of floods, and other practices of care for nature -- can be explained and systematized by the PHS proposed here. Through popular protagonism, this knowledge has the potential to strengthen communities and the health system in the face of the challenges imposed by climate emergencies. The organization of territorial-based collectives that can monitor threats to life in an agile and context-specific way during climate emergencies can have its work enhanced when articulated with intersectoral public policies [12,27,28]. These insights draw upon specific territorial experiences in Brazil, but the principles and frameworks described are generalizable to diverse sociocultural and environmental contexts, offering relevance and support to other settings facing climate-related challenges and vulnerabilities.

### 3.3 Final considerations

Popular Health Surveillance is not only a monitoring tool, but a strategy that can be mobilized across diverse contexts for political resistance and for the promotion of socio-environmental, cognitive, and health justice. In the face of intensifying climate crises, this approach is essential for strengthening communities’ capacity to adapt, integrate diverse forms of knowledge, and demand more inclusive and democratic public policies. In a scenario of global climate emergency, investing in Popular Health Surveillance means not only ensuring the health and well-being of vulnerable populations, but also redefining the paradigms of environmental and health governance.

Recognizing and strengthening the role of communities in the defense of their territories is an essential step towards building a fairer, more sustainable, and more solidary future. It is necessary to reinforce partnerships among governments, research institutions, social movements, and communities in order to advance inclusive public policies that that acknowledge and support community-based health surveillance initiatives. Popular Health Surveillance can serve as a powerful strategy to confront the challenges posed by climate emergencies, promoting health, environmental justice, and citizen participation. By integrating technical and popular knowledge, this approach not only enhances the resilience of communities but also contributes to construction of more democratic, inclusive, and sustainable health systems. In a world increasingly affected by climate emergencies, Popular Health Surveillance is an urgent necessity to safeguard the well-being of present and future generations.

**This article is one of the products of the research developed and supported by**

This work received technical support and funding from the Alliance for Health Policy and Systems Research and the WHO Health Emergencies Programme. The Alliance is able to conduct its work thanks to the commitment and support from a variety of funders. These include our long-term support from the Swedish International Development Cooperation Agency (Sida) and the Norwegian Agency for Development Cooperation (Norad), as well as designated funding for specific projects within our current priorities. For the full list of Alliance donors, please visit https://ahpsr.who.int/about-us/funders. The international funders had no role in study design, data collection and analysis, decision to publish, or preparation of the manuscript. The research also received financial support from Coordination of Health Surveillance and Reference Laboratories of the Presidency of Fiocruz, Fiocruz Ceará - Participatory in Health and Ecology of Knowledge and SERPOVOS.
